# Detection and Molecular Characterization of Canine Alphacoronavirus in Free-Roaming Dogs, Bangladesh

**DOI:** 10.3390/v14010067

**Published:** 2021-12-30

**Authors:** Mohammad Enayet Hossain, Ariful Islam, Shariful Islam, Md Kaisar Rahman, Mojnu Miah, Md Shaheen Alam, Mohammed Ziaur Rahman

**Affiliations:** 1 International Centre for Diarrhoeal Diseases Research, Bangladesh (icddr,b), Dhaka 1212, Bangladesh; enayet.hossain@icddrb.org (M.E.H.); mojnu.miah@icddrb.org (M.M.); shaheen.alam@icddrb.org (M.S.A.); mzrahman@icddrb.org (M.Z.R.); 2 EcoHealth Alliance, New York, NY 10001-2320, USA; sharifdvm51@gmail.com (S.I.); kaisar.kaif@gmail.com (M.K.R.); 3 Centre for Integrative Ecology, School of Life and Environmental Science, Deakin University, Burwood, VIC 3216, Australia; 4 Institute of Epidemiology, Disease Control and Research (IEDCR), Mohakhali, Dhaka 1212, Bangladesh

**Keywords:** Bangladesh, canine, coronavirus, epidemiology, zoonotic

## Abstract

Canine coronavirus (CCoV) is widespread among the dog population and causes gastrointestinal disorders, and even fatal cases. As the zoonotic transmission of viruses from animals to humans has become a worldwide concern nowadays, it is necessary to screen free-roaming dogs for their common pathogens due to their frequent interaction with humans. We conducted a cross-sectional study to detect and characterize the known and novel Corona, Filo, Flavi, and Paramyxoviruses in free-roaming dogs in Bangladesh. Between 2009–10 and 2016–17, we collected swab samples from 69 dogs from four districts of Bangladesh, tested using RT-PCR and sequenced. None of the samples were positive for Filo, Flavi, and Paramyxoviruses. Only three samples (4.3%; 95% CI: 0.9–12.2) tested positive for Canine Coronavirus (CCoV). The CCoV strains identified were branched with strains of genotype CCoV-II with distinct distances. They are closely related to CCoVs from the UK, China, and other CoVs isolated from different species, which suggests genetic recombination and interspecies transmission of CCoVs. These findings indicate that CCoV is circulating in dogs of Bangladesh. Hence, we recommend future studies on epidemiology and genetic characterization with full-genome sequencing of emerging coronaviruses in companion animals in Bangladesh.

## 1. Introduction

Coronavirus (CoV) is an enveloped, non-segmented, single stranded RNA virus classified under the family Coronaviridae. This family hosts several human and animal CoVs which affect both respiratory and enteric systems. Under the subfamily Coronavirinae, there are four genera: Alphacoronavirus (α-CoV), Betacoronavirus (β-CoV), Gammacoronavirus and Deltacoronavirus [1,2,3]. Human and other mammalian species generally become infected by all four genera of CoVs, while avian species only by Gammacoronavirus and Deltacoronavirus [4]. In the recent past, the world has faced CoV outbreaks belonging to Betacoronavirus (SARS-CoV-1, MERS-CoV, SARS-CoV-2 etc.) [5,6]. The current SARS-CoV-2 and previous SARS-CoV-1 and MERS-CoV zoonotic coronaviruses that have recently transferred from animal to human populations all belong to the β-CoVs [4]. CoVs are capable of causing, variably, severe intestinal, respiratory, neurologic, or systemic disease syndromes and finally death [4,7]. Recently SARS CoV-2 has been identified in dogs, lions, and tigers from different countries, inferring that they might have been transited from infected humans [8,9,10]. Beyond SARS CoV-2, Canine coronavirus (CCoV) belongs to α-CoV, and canine respiratory coronavirus (CRCoV) belongs to β-CoV, but both can infect dogs [3]. A study released on 20 May 2021 investigated samples collected between 2017 and 2018 from eight patients with pneumonia (seven of whom were children) in Malaysia and discovered a new coronavirus strain CCoV-HuPn-2018 [11]. Another sequence of CCoV strain Z19 (MZ420153) was found in a NCBI database which was detected in a Haitian human in 2017 [12]. If the association of CCoV with human disease is confirmed, it would become the eighth known coronavirus to cause human disease.

Based on spike protein amino acid sequences, CCoVs are classified into two distinct genotypes, named CCoV-I and CCoV-II [13]. CCoV-II can be further divided into CCoV-IIa and CCoV-IIb. CCoV type I is genetically more similar to feline coronavirus (FCoV) type I than to CCoV type II. On the other hand, FCoV type II evolved by heterologous recombination between CCoV type II and FCoV type I [14].

CCoV is considered to be responsible for gastrointestinal infection in dogs; more precisely, young puppies are most susceptible [15,16]. Usually, coronavirus replicates in enterocytes of the tip of the intestinal villus and causes a varying degree of enteritis and shedding of virus for a long period through feces [17,18]. It leads to lethargy, anorexia, vomiting, diarrhea, dehydration, and lymphopenia lasting for 1–2 weeks in dogs [15,16].

CCoV is highly contagious but sometimes it causes asymptomatic infection and occasionally causes death in young puppies, particularly co-infected with other pathogens such as canine parvovirus [13,16]. Hence, the symptoms of infected dogs depend on the age of the animals, level and type of infected organism, and immunity developed by maternal antibodies [13]. The virus can cause respiratory distress and has been previously detected from respiratory samples [19,20]. CCoV has been identified at high prevalence in kenneled dogs than in other types of dog. The prevalence was not strongly associated with age, sex, breed, and health condition [21]. However, the infection depends on dog management practices [22].

The exact number of dogs in Bangladesh is still unknown. But it was found that 64% of all dogs in Bangladesh are stray dogs and another 30% were estimated as unrestricted neighborhood dogs [23]. There are approximately 14 dogs/km^2^ and the human–dog ratio is 120:1 [24]. Another study estimated that there are a total of 18,585 free-roaming/stray dogs in Dhaka city (52 dogs/km^2^) where the human–dog ratio is 828:1 [25]. Stray dogs roam around public places and search for food in wastage or garbage. These stray dogs have frequent interaction with humans, especially with children who live in slums [23]. Distribution of street dogs and their foraging behavior are closely linked with the cultural and socioeconomic conditions of humans [25]. These free-roaming dogs can be a source of serious health hazards for humans. Transmission of emerging zoonotic pathogens like SARS-CoV and MERS-CoV from animal host to human has been a concern worldwide [26,27]. It is crucial to detect and characterize the novel virus circulating animal host and stop spillover to humans at high interfaces and to prevent future epidemics and pandemics by disease X. Coronavirus [21,28,29], Filovirus [30], Flavivirus [31,32], and Paramyxoviruses [33,34,35] have been identified in dogs in other countries, but Bangladesh lags behind in this aspect. Therefore, it is crucial to know the viral diversity in dog populations, molecular characterization, and phylogenetic analysis of the circulating strains. The study aimed to detect and characterize the known and novel Corona, Filo, Flavi, and Paramyxoviruses in free-roaming dogs in Bangladesh.

## 2. Materials and Methods

### 2.1. Study Location and Duration

**Figure 1:**
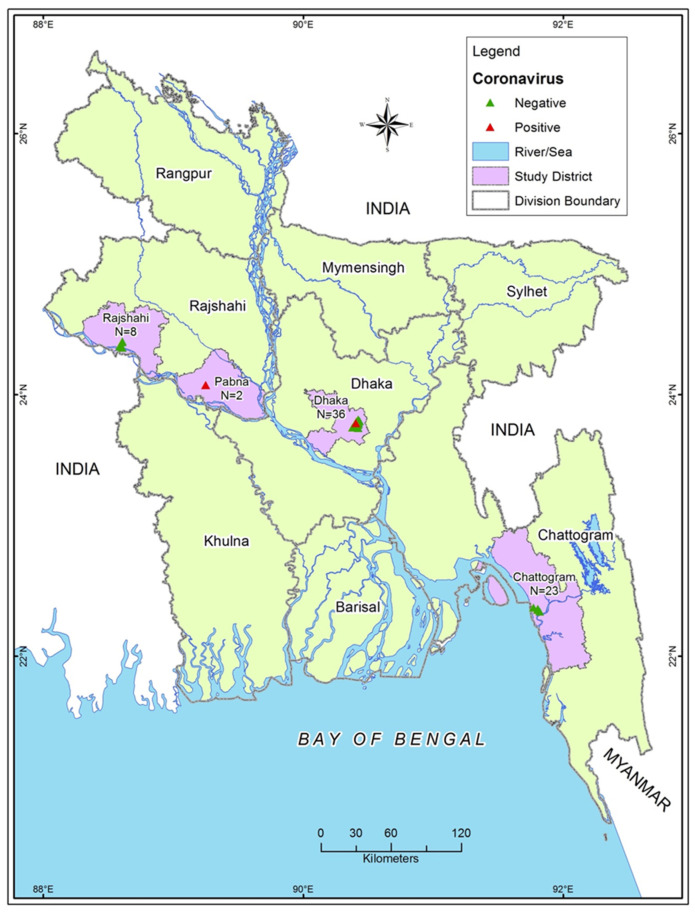
Sampling sites and sample numbers from 4 study districts.

We conducted a cross-sectional study in four districts of Bangladesh (Chattogram, Dhaka, Pabna, and Rajshahi) during the dry season (November to April) and wet season (May to October) between 2009–10 and 2016–17 (Figure 1). We sampled a total of 69 dogs from the city corporation area of the study districts.

### 2.2. Dog Capturing, Sample Collection and Processing, Data Collection

We captured and restrained stray dogs (have no owner) and sheltered dogs (have an owner but can mix with stray dogs) according to the safe animal capture and sampling protocol of USAID PREDICT (PREDICT One Health Consortium 2016) [36,37,38]. Physical restraining was accomplished with full personal protective equipment including nitrile gloves, N95 mask, goggles, Tyvek type suits, and specialized equipment such as leather gloves and rabies-poles [36]. We collected oropharyngeal swab and rectal swab samples from each dog. Samples were kept in two aliquots in 500 μL trizol or lysis buffer and 500 μL viral transport media (VTM). Immediately after sample collection, we released the animals at their capturing sites. All samples were stored in liquid nitrogen in the field and transferred to laboratory for storage in −80 °C freezer until further analysis. The team collected the necessary information for individual dogs such as age, sex, breed, health condition (healthy or sick), body condition score (BCS), and physical examination findings using a structured multiple-choice questionnaire. To determine age, we checked the dentition, physical growth, and external genital organs [36,39,40,41]. For BCS, we inspected the visible body fat coverage, following International Companion Animal Management guidelines and categorized as poor (emaciated), fair (underweight) and good (normal, overweight or obese) [42].

### 2.3. Laboratory Analysis

We tested the samples for Corona, Filo, Flavi, and Paramyxovirus. After extraction of RNA, we synthesized cDNA using the SuperScript III RNase H Reverse transcriptase kit (Invitrogen SA, Spain/Portugal, Barcelona, Spain). Filovirus, Flavivirus, and Paramyxovirus were screened according to the procedure described by Moureau and Temmam [43], Zhai and Palacios [44] and Tong and Chern [45] protocols, respectively.

Two conventional PCR techniques were used targeting non-overlapping fragments of the orf1ab to identify known and novel coronaviruses [46,47]. We used a heminested PCR to amplify the highly conserved region of the RNA-dependent RNA polymerase (RdRp) gene (~434 bp) corresponding to nucleotides (NTs) 14,123–14,556 in the canine coronavirus strain 2020/7 genome (MT906865) following Watanabe et al. [47] with some modifications. Quan et al. [46] amplified a ~328 bp fragment of nsp14 gene corresponding to NTs 17,282–17,609 in the canine coronavirus strain 2020/7 genome (MT906865).

According to the traditional Sanger dideoxy sequencing, amplified products of the expected size were sequenced and further edited manually in Geneious Pro (version 9.1.3, Biomatters, Auckland, New Zealand). All gene sequences generated in the study were submitted to the GenBank database.

We searched for highly similar sequences to our sequences in GenBank using the Basic Local Alignment Search Tool (BLAST). Sequences that have >90% similarities with a sequence already in GenBank are considered as a known virus [48]. RdRp and Nsp14 gene sequences of different reference strains of Feline coronavirus (FCoV), porcine transmissible gastroenteritis virus (TGEV), and canine coronavirus (CCoVs) were retrieved from the National Center for Biotechnology Information (NCBI). All the retrieved sequences were aligned with Bangladeshi CCoV sequences by subjecting to ClustalW multiple sequence alignment using BioEdit 7.1.3 program [49]. Evolutionary relationships were inferred by using the Maximum Likelihood method based on the Hasegawa-Kishino-Yano model [50] from 1000 bootstrap values. The evolutionary distances were computed using the Kimura 2-parameter method, and evolutionary analyses were conducted in MEGA 7.

To better visualize the genetic relatedness of viruses, a median-joining phylogenetic network was constructed based on the partial RdRp gene by using NETWORK version 10.1 with epsilon set to 0 (www.fluxus-engineering.com, accessed on 13 December 2021). To generate a phylogenetic network, CCoV and FCoV sequences were subjected to ClustalW multiple sequence alignment using BioEdit 7.1.3 program [49] and a haplotype data file was generated using the DNA sequence polymorphism analysis tool DnaSP (http://www.ub.edu/dnasp/, accessed on 13 December 2021).

### 2.4. Statistical Analysis

We used Microsoft Office Excel 2013 (Redmond, WA, USA) for data entry and management while STATA-13 (StataCorp 4905, Lakeway Drive, College Station, TX 77845, USA) was used for data analysis. A dog was considered positive if any of the 3 samples from each dog was tested positive in either protocol of PCR for CoV [46,47]. Descriptive analysis was done to summarize frequency and percentage with 95% confidence interval (CI).

## 3. Results

None of the samples were found positive for Filo, Flavi and Paramyxovirus. Among 69 street dogs, 4.3% (*n* = 3; 95% CI: 0.9–12.2) were positive for coronavirus. CoV positive samples were from Dhaka (*n* = 1, 2.8%; 95% CI: 0.07–14.5) and Pabna (*n* = 2; 100%; 95% CI: 15.8–100), sampled in dry season (*n* = 3; 18.8%; 95% CI: 4.0–45.6). All three positive dogs were adult (*n* = 3; 23.1%; 95% CI: 5.0–53.8), two were female (*n* = 2; 6.1%; 95% CI: 0.7–20.2) and one was male (*n* = 1; 2.8%; 95% CI: 0.07–14.5). The positive dogs had fair (*n* = 1; 3.5%; 95% CI: 0.08–17.8) and poor body condition (*n* = 2; 9.1%; 95% CI: 1.1–29.1) (Table 1).

The three positive samples were from rectal samples of dogs. The samples tested positive in both protocols used in this study. Therefore, we obtained six sequences from three rectal swab samples. All gene sequences generated in the study were submitted to the GenBank database under the accession numbers MT083414, MT083415, MT083436, MT064650, MT064651, and MT064809. The sequences have 97.24% similar identity with canine coronaviruses.

**Figure 2:**
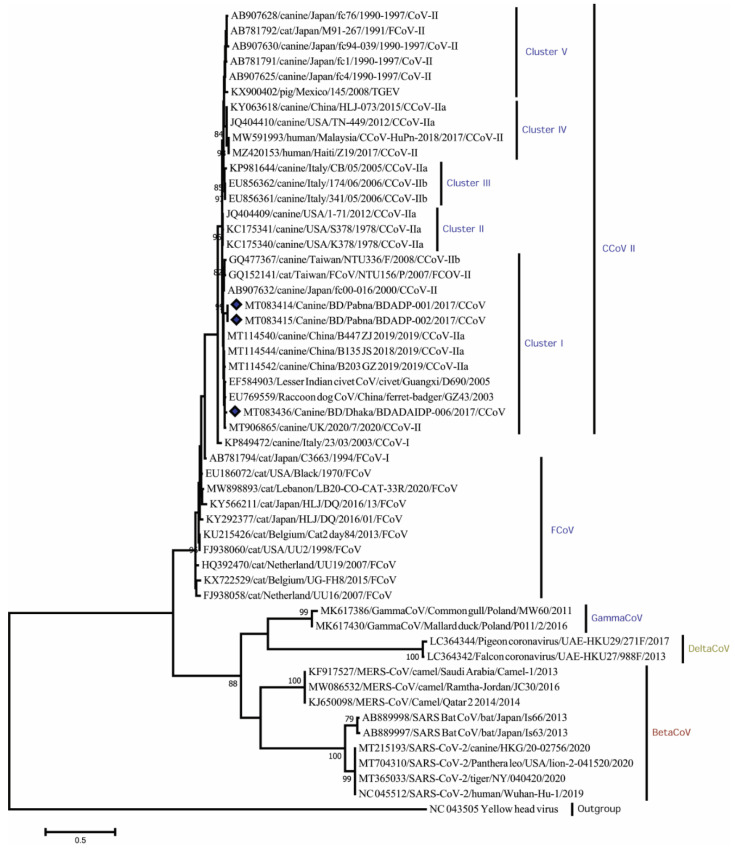
Maximum Likelihood phylogenetic tree based on the partial RdRp sequence of Coronaviridae. CCoV strains in this study are indicated by rhombus (blue colored). The tree was calculated using the Maximum Likelihood method with the Kimura 2-parameter distance model. The percentages of replicate trees (>70%) in which the associated taxa clustered together in the bootstrap test (1000 replicates) are shown next to the branches. RdRp, RNA dependent RNA polymerase; CCoV, canine coronavirus; CCoV-I, canine coronavirus type I; CCoV-II, canine coronavirus type II; CCoV-IIa, canine corona-virus subtype IIa; CCoV-IIb, canine coronavirus subtype IIb; FCoV, feline coronavirus.

Phylogenetic analysis using partial RdRp genes showed that Bangladeshi CCoV strains are branched in cluster I of canine coronavirus type II. All CCoV strains isolated from dogs: BDADP-01, BDADP-02, and BDADAIDP-006 belong to the CCoV-II group. Of them, two CCoV-II strains BDADP-01, BDADP-02 collected from Pabna district, branched in cluster I. Another CCoV-II strain BDADAIDP-006 collected from Dhaka, formed a close cluster with CCoV strain 2020/7 (MT906865), Raccoon dog CoV strain GZ43/03 (EU769559), civet coronavirus strain D690/05 (EF584903), and CCoV strain B135_JS_2018 (MT114544), shared >98% nucleotide identities (Figure 2). Bangladeshi CCoV strains exhibited 96.3–100% nucleotide identities. The sequence comparison of the partial RdRp gene revealed amino acid homology of 100% among three Bangladeshi CCoV strains and with other type II CCoVs within cluster I. Two CCoV-II strains isolated from humans in Malaysia (MW591993) [10] and Haiti (MZ420153) showed 93.77–94.5 nucleotide identities and 100% amino acid homology with Bangladeshi CCoVs detected in dogs.

**Figure 3:**
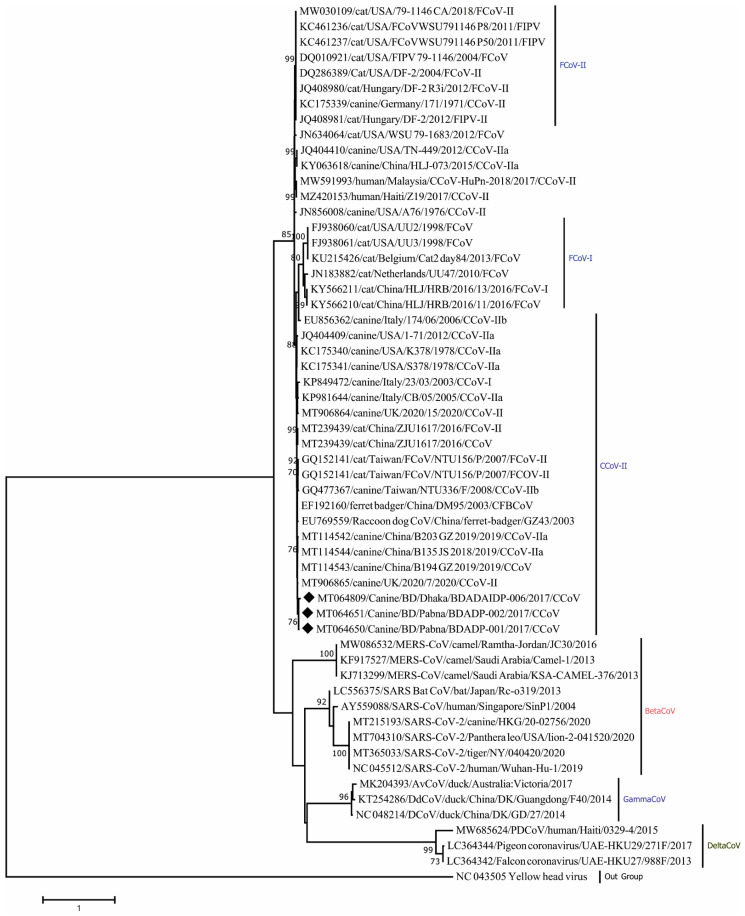
Maximum Likelihood phylogenetic tree based on the partial nonstructural protein 14 (Nsp14) sequence of Coronaviridae. CCoV strains in this study are indicated by rhombus (blue colored). Canine CoV, feline CoV, Betacoronavirus, Gammacoronavirus, Deltacoronavirus sequences are shown in the phylogeny. The tree was calculated using the Maximum Likelihood method with the Kimura 2-parameter distance model. The percentages of replicate trees (>70%) in which the associated taxa clustered together in the bootstrap test (1000 replicates) are shown next to the branches.

Phylogenetic analysis revealed that Nsp14 genes of three CCoV strains clustered closely within themselves and with other CCoV-II strains (Figure 3). Amino acid sequence comparison unveiled homologies of 97.94–98.97% (K176R, S185A/T variations) with other CCoV-II strains as well as 98.97% (S185A/T variation) with CCoV-II strains isolated from humans.

**Figure 4:**
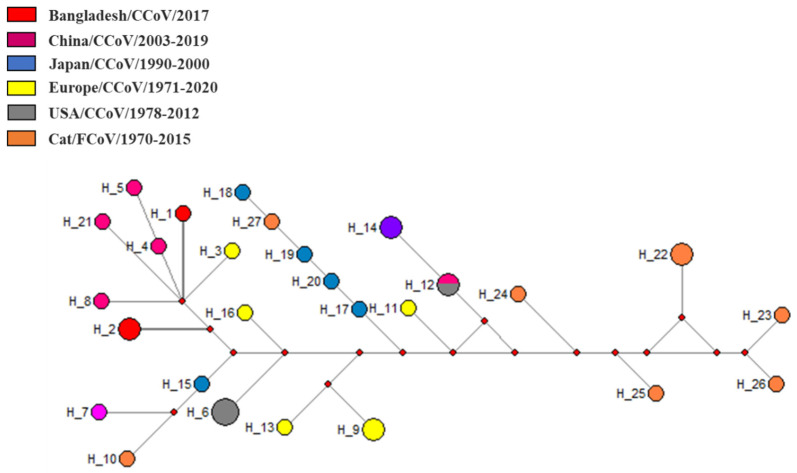
Median-joining phylogenetic network of Coronaviridae. The median-joining network was constructed from the partial RdRp sequences. Each unique sequence is represented by a circle sized relative to its frequency in the dataset. Isolates are colored according to the location. RdRp, RNA-dependent RNA polymerase.

Median-joining phylogenetic network analysis demonstrated that the studied CCoVs strains formed separate nodes within other CCoV-II strains from China and Europe which confirmed the presence of type II CCoV strains in Bangladesh (Figure 4). Network analysis findings support the result of the evolutionary phylogeny analysis.

## 4. Discussion

To the author’s knowledge, we screened free-roaming dogs for Corona, Filo, Flavi, and Paramyxovirus for the first time in Bangladesh. We detected CCoV in 4.3% of tested samples which is lower than reports from other countries such as Italy, China, Turkey, England, Japan, Greece, and Portugal [29,51,52,53,54]. Studies conducted in China have also reported variation of CCoV prevalence in different regions, for example, 26% in Beijing [55], 28.36% in Heilongjiang [56], and 23.94% in Guangdong, Zhejiang, Jiangsu, and Anhui [29].

Though we collected and tested specimens from four different locations, CCoV was detected in Dhaka and Pabna districts only. We investigated the association of CCoV infection with age, sex, ownership status, BCS, and health condition but did not find any significant relations. A similar finding was also reported from Japan [21]. A higher prevalence of CCoV was found in healthy dogs aged >1 year while prevalence was greater among diarrheic dogs in younger animals aged <1 year [21]. Previous studies reported that both pup and adult dogs can be infected by CCoV and develop gastro-enteritis [57].

Besides, the dog population in Bangladesh may carry CCoV asymptomatically [13,16]. In some cases, they might have been infected previously but recovered, still shedding CCoV for several months [18,22]. We did not find any severe diarrheic dogs positive for CCoV infection which might be due to genomic mutation in the host, i.e., genomic recombination with other coronaviruses which might lead to shifting and drifting in order to change their pathogenicity and symptoms [21].

In the current study, we detected type II CCoV strains which have already been detected in dogs [17,21,22,54]. The conducted analysis demonstrated that newly identified Bangladeshi CCoV strains, BDADP-001, BDADP-002, and BDADAIDP-006 were closely clustered with CCoV-II strains from badger, civet, and dogs from China and UK. Phylogenetic, median-joining network tree and pairwise distance analyses revealed nucleotide similarities of Bangladeshi CCoV strains to FCoV strain NTU156 isolated from cats in Taiwan, CCoV strains responsible for human infection in Malaysia and Haiti, and a TGEV strain from pigs. These findings suggest a long history of the transmission cycle from related species in different geographical locations and the recombinant nature of CCoV [13]. The history of human infection with the CCoV strain warrants the threat of zoonotic potentials and CCoV could become the eighth known coronavirus to cause human disease [10].

In this study, we confirmed the presence of CCoVs in dogs by sequencing only a short highly conserved region of the RdRp and NSP14 genes. We could not perform recombinational analysis due to a lack of complete genome or at least full gene (RdRp/NSP14) sequence. Previous studies suggest natural interspecies recombination of FCoVs and CCoV as well as interspecies transmission of CCoVs [58,59,60]. Besides, several studies reported the human infection of CCoV and/or novel recombinant CCoV in multiple countries with acute respiratory symptoms [11,12,61,62]. Gonzalez, Gaelle, et al. claim that dogs might act as “mixing vessels” in which novel viruses with pandemic potential could emerge [63]. Since canines, felines and humans are highly exposed to each other, interspecies transmission of emerging viruses like CCoVs and FCoVs is possible.

Our conducted analyses revealed 96.3–100 sequence homology among Bangladeshi CCoV-II strains and suggest local evolution of the viruses. Phylogenetic analysis of partial RdRp gene confirmed that Bangladeshi CCoV strains were distantly related (72–75% nt identities) to other Alphacoronavirus species, such as HCoV (NL63), bat CoVs isolated in Kenya, Mozambique, USA, and rodent CoV in China (data not shown). Bangladeshi CCoVs showed ~58% sequence homology with SARS-CoV-2 strain 20-02756/2020 isolated from a dog in Hong Kong during the current pandemic outbreak in 2020 (Figure 2).

Coronaviruses have a high potential for the emergence of new strains as with SARS CoV-1 and 2, with genomic mutation and recombination. It has also been assumed that CCoV is genomically much more complex which might increase the likelihood of novel virus emergence within dogs [13]. Natural infection of SARS-CoV-2 was found in domestic dogs. The first report of a dog infected by SARS-CoV-2 was found in a Pomeranian dog in Hong Kong [8]. The owner of this dog also tested positive for COVID-19 a few days later [8]. Later, several dogs were found to be infected without showing any symptoms in Hong Kong. All of these dogs had a history of mutual living alongside infected humans [8,55]. Another dog was infected by its owner in The Netherlands [64].

The main limitation of our study is the small sample size. Considering the lack of data from Bangladesh in this regard, the present study primarily focused on the detection and molecular characterization of circulating viral strains in Bangladesh.

## 5. Conclusions

CCoV has substantial sequence similarity with FCoV. Sharing a habitat with canine and feline species, especially cats, increases the probability of the emergence of novel strains of coronavirus across the species. To understand in further detail the molecular epidemiology and ecology of important viruses in the dog population of Bangladesh, more carefully planned and larger similar studies are required in the future.

## Figures and Tables

**Table 1:** Distribution of canine coronavirus in Dogs, Bangladesh.

Variables	Categories	N	CCoV Positive *n* (%)	95% CI
District	Chattogram	23	-	-
	Dhaka	36	1 (2.8)	0.07–14.5
	Pabna	2	2 (100)	15.8–100
	Rajshahi	8	-	-
Sampling time	2009–2010	53	-	-
	2016–2017	16	3 (18.8)	4.0–45.6
Sampling season	Dry	16	3 (18.8)	4.0–45.6
	Wet	53	-	-
Sex	Male	36	1 (2.8)	0.07–14.5
	Female	33	2 (6.1)	0.7–20.2
Age	1–1.5 years	32	-	-
	1.6–3 years	24	-	-
	>3 years	13	3 (23.1)	5.0–53.8
Type of ownership	Stray dog	58	2 (3.5)	0.4–11.9
	Sheltered dog	11	1 (9.1)	0.2–41.2
Health condition	Sick	13	1 (7.7)	0.2–36.0
	Apparently healthy	56	2 (3.6)	0.4–12.3
BCS	Poor	22	2 (9.1)	1.1–29.1
	Fair	29	1 (3.5)	0.08–17.8
	Good	18	-	-

## Data Availability

The data that have been used in this study will be available from the corresponding author upon requests.
